# The Effects of *Trifolium pratense* L. Sprouts’ Phenolic Compounds on Cell Growth and Migration of MDA-MB-231, MCF-7 and HUVEC Cells

**DOI:** 10.3390/nu12010257

**Published:** 2020-01-19

**Authors:** Małgorzata Zakłos-Szyda, Grażyna Budryn

**Affiliations:** 1 Institute of Molecular and Industrial Biotechnology, Faculty of Biotechnology and Food Sciences, Lodz University of Technology, Stefanowskiego 4/10, 90-924 Lodz, Poland; 2 Institute of Food Technology and Analysis, Faculty of Biotechnology and Food Sciences, Lodz University of Technology, Stefanowskiego 4/10, 90-924 Lodz, Poland; grazyna.budryn@p.lodz.pl

**Keywords:** *Trifolium pratense* L., isoflavones, breast cancer, migration, estrogen receptors

## Abstract

Uncontrolled growth and migration and invasion abilities are common for cancer cells in malignant tumors with low therapeutic effectiveness and high mortality and morbidity. Estrogen receptor β (ERβ), as a member of the nuclear receptor superfamily, shows potent tumor suppressive activities in many cancers. Phytoestrogens’ structural resemblance to 17 β-estradiol allows their binding to ERβ isoform predominantly, and therefore, expression of genes connected with elevated proliferation, motility and invasiveness of cancer cells may be downregulated. Among polyphenolic compounds with phytoestrogenic activity, there are isoflavones from *Trifolium pratense* L. (red clover) sprouts, containing high amounts of formononetin and biochanin A and their glycosides. To determine the source of the most biologically active isoflavones, we obtained four extracts from sprouts before and after their lactic fermentation and/or *β*-glucosidase treatment. Our previous results of ITC (isothermal titration calorimetry) modelling and a docking simulation showed clover isoflavones’ affinity to ERβ binding, which may downregulate cancer cell proliferation and migration. Thus, the biological activity of *T. pratense* sprouts’ extracts was checked under in vitro conditions against highly invasive human breast cancer cell line MDA-MB-231 and non-invasive human breast cancer cell line MCF-7 cells. To compare extracts’ activities acquired for cancer cells with those activities against normal cells, as a third model we choose human umbilical vein endothelial cells (HUVEC), which, due to their migration abilities, are involved in blood vessel formation. Extracts obtained from fermented sprouts at IC_0_ dosages were able to inhibit migration of breast cancer cells through their influence on intracellular ROS generation; membrane stiffening; adhesion; regulation of MMP-9, N-cadherin and E-cadherin at transcriptional level; or VEGF secretion. Simultaneously, isolated phenolics revealed no toxicity against normal HUVEC cells. In the manuscript, we proposed a preliminary mechanism accounting for the in vitro activity of *Trifolium pratense* L. isoflavones. In this manner, *T. pratense* sprouts, especially after their lactic fermentation, can be considered a potent source of biological active phytoestrogens and a dietary supplement with anti-cancer and anti-invasion properties.

## 1. Introduction

Cancer cells’ uncontrolled growth, and their migration and invasion abilities, are commonly found in malignant tumors. According to the World Health Organization, breast cancer is the most frequent malignant tumor among women, impacting 2.1 million women each year (WHO, www.who.int/cancer). Due to low therapeutic effectiveness, breast cancer is the main cancer-related cause of female deaths, with the level of 15% of mortality worldwide. Among important therapeutic targets the control of growth, the invasion and the metastasis of cancer cells have been searched. Estrogen receptor β (ERβ), as a member of the nuclear receptor superfamily, shows potent tumor suppressive activities in many cancers [1]. Estrogens such as 17 β-estradiol work as natural ligands for estrogen receptors (ER)—proteins belonging to the nuclear receptor superfamily. There are two estrogen receptor isoforms, ERα and ERβ, which are localized in the cytoplasm and in the nucleus. The 17 β-estradiol molecule passively enters the cell through the plasma membrane, and after binding with ERs initiates different cellular processes, i.e., proliferation, differentiation and survival, via regulation of genes transcription after binding to the estrogen-responsive element (ERE) on the promotors of the ER target genes site, and modification of chromatin structure [2]. Despite the classical or genomic action of steroid receptors, they possess also non-genomic rapid activity via upstream interactions with signaling or scaffold proteins controlling cell cycle, proliferation, migration or nuclear exclusion of steroid receptors [3,4]. Through the direct interaction of ERα with proteins, activation of the Src- and PI3-kinase-dependent pathways occurs, which induces DNA synthesis and cytoskeleton changes in breast cancer MCF-7 cells in the absence of 17 β-estradiol. Because ER location regulates the estradiol signaling output, specific targeting of the interaction between estrogen receptors and signaling effectors (like Src family tyrosine kinases involved in signaling transduction pathways) or nuclear export receptors (exportin/Crm1 protein involved in ER export form nuclei) antagonizes the proliferative rate of breast cancer cells [3,4]. Both ERα and ERβ proteins are expressed not only in many normal tissues, but also in cancers with motility and invasive properties [1]. Data analysis showed that almost 75% of all breast cancers express ERα isoform and its activation leads to enhanced cells proliferation, while ERβ induction reduces cell proliferation and suppresses tumorigenesis [5]. What is more, ERβ has been described as a dominant negative regulator of estrogen signaling, because formation of heterodimers with ERα represses ERα mediated transcription.

Because estrogens significantly participate in growth, mineralization and remodeling of bone tissue, and regulate lipid metabolism in the liver, their deficiency negatively effects these processes [6]. Among food components, there are many phytochemicals showing estrogenic potency, which are known as phytoestrogens. These plant-derived polyphenols structurally and functionally mimic 17 β-estradiol by binding to the estrogen receptors—preferentially to the ERβ isoform, leading the prevention of ERα growth promotion activity [7]. Among polyphenolic compounds with phytoestrogen activity, there are isoflavones which can be found in food, especially in Asian countries. The consumption of isoflavones in Asia (≈100 mg/day with soy foods), which is significantly higher than in non-Asian countries (≈3 mg/day), is strongly connected with lower breast cancer incidence. Thus, it is not surprising that there is need to find estrogen replacement therapy for women at menopause and in the postmenopausal period to stimulate the growth of bone tissue and reduce the risk of atherosclerosis without estrogen-induced tumorigenesis [8]. Because isoflavones can accumulate at 100-fold higher concentrations than endogenous estrogens, they make strong impacts on signaling and processes with or without mediation by the estrogen receptor [9]. Isoflavones are synthesized mainly by leguminous plants, and one of the richest sources of these compounds, next to the soybean, is red clover (*Trifolium pratense* L.) [10]. *T. pratense* is a commonly found perennial herb, present in herbal medicines as dried floral blossoms used for skin treatment and management of menopausal complaints; however, its sprouts are largely unused [11]. Red clover displays high contents of isoflavone aglycons—biochanin A and formononetin, which are 4′-*O*-methylated plant precursors to genistein and daidzein. These methoxylated aglycones are quickly absorbed into the bloodstream from the gastrointestinal tract without the need for their hydrolysis with bacterial glycosidases, in contrast to glycosides [10].

Our previous results of isothermal titration calorimetry (ITC) modelling and docking simulation with red clover sprouts’ isoflavones clearly showed their higher affinity to ERβ than ERα, indicating their great potential to advantageously activate ERβ without leading to breast cancer cells’ proliferation [12]. What is more, further comparable studies revealed red clover main isoflavones’ ability to form spontaneous bond with Lys_18_ located in ATP binding site of actin through formation of π–π-type linkages [13]. Thus, the presence of isoflavones may interrupt globular monomer of actin (G-actin) polymerization, rapid reorganization and formation of filaments (F-actin), which further may reduce cancer cell migration, metastasis and cells survival. What is more, observed interactions of actin with biochanin A were endothermic leading to conformational rearrangement in the actin molecule, which may influence also transcriptional regulation controlled by actin allosteric effects. Taking these observations into account, the main goal of our studies was to check the biological activity of isoflavones isolated from *T. pratense* sprouts. To determine the source of the most biologically active phenolics, we obtained four extracts from sprouts before and after their lactic fermentation and/or enzymatic glucose cleavage. As cellular models were chosen, highly invasive human breast cancer cell line MDA-MB-231 and non-invasive human breast cancer cell line MCF-7 were the picks. These cell lines are well-established in vitro models for evaluating estrogen-responsive or estrogen-independent chemicals. To compare activities for cancer cells with normal cells, as a third model we chosen human umbilical vein endothelial cells (HUVEC), which due to their migration abilities are involved in blood vessel formation. Biological studies determined *T. pratense* polyphenols’ phytoestrogen potential—as ligands for ER-involved cellular signaling transducers and selected genes transcription regulators (*ERα/β*, *MMP2/9*, *E-cadherin/N-cadherin*, *VEGF*), and as agents able to influence the viability, intracellular oxidative stress, migration, adhesion and membrane fluidity of cells.

## 2. Materials and Methods

### 2.1. Chemicals and Reagents

All chemicals used, if not stated otherwise, were obtained from Sigma-Aldrich (St. Louis, MO, USA). All cell culture reagents were obtained from Life Technologies (Carlsbad, CA, USA). Tissue culture plastics were supplied by Greiner Bio-One GmbH (Frickenhausen, Austria). All the experimental measurements, if not stated otherwise, were performed using the Synergy 2 BioTek microplate reader (Winooski, VT, USA). Microscopic observations were performed using a fluorescent microscope—Nikon TS100 Eclipse (Tokyo, Japan) under ×200 magnification, or the MDG41 Leica (Leica Microsystems, Wetzlar, Germany) under ×10 magnification, if not stated otherwise.

### 2.2. Preparation of Red Clover Extracts and Phenolic Compounds Determination

Red clover seeds (*Trifolium pratense* L.) were obtained from FN Granum (Wodzierady, Poland). Seed germination and fermentation carried out with *Lactobacillus casei* 0979 strain were performed as described previously [10,12]. Lactic acid fermentation was performed to increase the concentration of phenolics and to convert some of the glycosides into aglycones. Sprouts and fermented sprouts were additionally hydrolyzed with *β*-glucosidase. Four preparations of isoflavones were obtained by freeze-drying of sprouts, extraction of dried sprouts in water (1:10, m/m) and freeze-drying of extracts, described as: clover treated with UVB (CU) (sprouts’ phenolic extract), clover/UVB/H (CUH) (sprouts’ phenolic extract after hydrolysis with β-glucosidase), clover/UVB/F (CUF) (fermented sprouts’ phenolic extract) and clover/UVB/F/H (CUFH) (fermented sprouts’ phenolic extract after hydrolysis with β-glucosidase). For biological activity assays, preparations were dissolved in a phosphate buffer solution (PBS)/dimethyl sulfoxide (DMSO) (1:1 *v*/*v*) solution at a concentration of 300 mg of extract/mL. The contents of isoflavones were determined as described previously by Budryn et al. [12] via LC-ESI-MS analysis of isoflavones with the use of a Shimadzu (Kyoto, Japan) liquid chromatograph equipped with a SPD M20A diode array detector and quadrupole m mass spectrometer 2020 with an ion source of electrospray type (ESI) in positive scan mode with Kinetex C18 column after elution and MS identification. The content of isoflavones was calculated on dry mass and presented as concentration of particular isoflavones, as well as total free aglycones and total isoflavones (Table 1).

### 2.3. Cell Culture and Exposure Conditions

Human breast cancer MCF-7 and MDA-MB-231 cell lines were grown in DMEM with 10% fetal bovine serum (FBS) medium supplemented with 100 U/mL penicillin, 100 µg/mL streptomycin and 25 µg/mL amphotericin B. Human umbilical endothelial cells (HUVEC) were grown in RPMI with 20% fetal bovine serum (FBS) medium supplemented with 150 µg/mL endothelial growth factor, 100 U/mL penicillin, 100 µg/mL streptomycin and 25 µg/mL amphotericin B. The cells were maintained at 37 °C in a humidified incubator containing 5% CO_2_. Cells were incubated with tested extracts for 24 and 48 h in starving medium (for migration studies). Tested extracts were obtained by dissolving sprouts extracts in a PBS/DMSO (1:1 *v*/*v*) at concentration 300 mg/mL and were further diluted with culture medium. The extract’s concentrations used in biological studies are presented in the descriptions of the tests carried out.

### 2.4. Cell Viability

**Figure 1:**
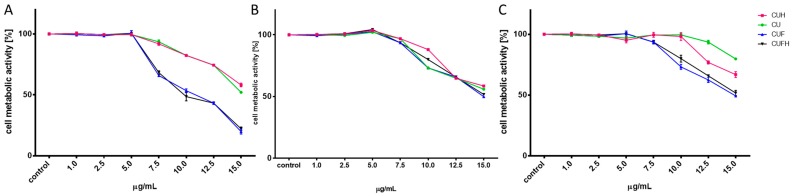
The influence of *T. pratense* extracts on MDA-MB-231 (**A**), MCF-7 (**B**) and HUVEC (**C**) cells’ metabolic activity determined by PrestoBlue assay after 48 h exposure to CU (sprouts polyphenolic extract), CUH (sprouts polyphenolic extract after hydrolysis), CUF (fermented sprouts polyphenolic extract) and CUFH (fermented sprouts polyphenolic extract after hydrolysis). Control cells were not exposed to any compound but the vehicle; values are means ± standard deviations from at least three independent experiments (*n* ≥ 16).

Cells were seeded into 96-well plates at 10^4^ cells per well in complete medium and grown for 24 h; then, medium was changed into starving medium (with 0.1% FBS), and cells were incubated in the presence of the studied extracts diluted in culture medium for either 48 h. Cells viability was quantified with PrestoBlue reagent according to the manufacturer’s instructions by measuring the fluorescent signal at F530/590 nm. The obtained fluorescence values were used to calculate cell viability expressed as the percentage of the viability of the untreated control cells (cells treated with equal volume of the vehicle instead of the preparation). To evaluate the protective effect of preparations against oxidative stress, cells were pre-incubated with IC_0_ amounts (concentration by which the viability amounted 100%, Figure 1) of extracts for 48 h [14]. Then, to induce the oxidative stress condition, 500 µM *tert*-butylhydroperoxide (*t*-BOOH) was added for 2 h and cellular viability with PrestoBlue reagent was measured.

### 2.5. Detection of Intracellular Reactive Oxygen Species (ROS) Generation

To determine the effect of extracts on the intracellular generation of ROS after 48 h of treatment with extracts, cells were loaded with the DCFH-DA (dichloro-dihydro-fluorescein diacetate) dye at a final concentration of 10 µM for 30 min. The fluorescent signal at F485/530 nm was analyzed. The negative control contained only cells in culture medium (without FBS), while the positive control contained 500 µM *t*-BOOH.

### 2.6. Measurement of Mitochondrial Membrane Potential (MMP)

The MMP was assayed with JC-1 probe. After 48 h treatments with the extracts, the medium was changed and JC-1 (1 µg/mL) was added for 20 min [15]. Then, the cells were washed with serum-free medium and fluorescent signal at F485/530 nm was measured.

### 2.7. Phosphatidylserine Externalization and Membrane Permeabilization

To quantify the level of externalized phosphatidylserine on the outer membrane leaflet of apoptotic cells, the Annexin-V-FITC assay kit was used (Sigma-Aldrich). After 48 h treatment with extracts, cells were washed twice with phosphate buffer solution and incubated with annexin-V-FITC (final concentration 0.25 µg/mL) for 10 min. Annexin-V binding was measured by the change in fluorescence F485/530 nm. Membrane permeabilization caused by investigated compounds was measured with propidium iodide (PI). After cells’ treatments with extracts for 48 h, PI was added at to final concentration of 1 µg/mL. Intercalation of PI was monitored by the change of fluorescence F535/620 nm.

### 2.8. Cell Migration Assay

To determine the effects of extracts on cellular migration, Oris™ Cell Migration Assays were done according to the manufacturer’s protocol with modifications [16]. Briefly, cells were seeded on the 96-well plate with “stopper” barriers that create a central cell-free detection zone for cell migration experiments. After 24 h, cells were starved for additional 24 h, and then the stoppers were removed, allowing cellular migration into the standardized detection zone. After addition of tested compounds at the highest nontoxic concentrations, photos of the zones were taken at 0, 24 and 48 h intervals (MDG41, Leica). Migration of stimulated cells versus unstimulated cells was calculated according to the formula:

(1) $$migration\;\left[\%\right]=\frac{V_{t0}-V_{ti}}{Vk_{t0}-Vk_{ti}}\;\times \;100\%,$$

where: *V_t_*_0_ is area of wound in stimulated samples at time 0 (mm^2^); *V_ti_* is area of wound in stimulated samples at time 24 or 48 h (mm^2^); *Vk_t_*_0_ is area of wound in control samples at time 0 (mm^2^); *Vk_ti_* is the area of a wound in a stimulated sample at time 24 or 48 h (mm^2^). Wound healing determination was calculated according to the formula:

(2) $$wound\;healing\;\left[\%\right]=\frac{V_{t0}-V_{ti}}{Vk_{t0}-Vk_{ti}}\;\times \;100\%.$$

### 2.9. Adhesion Assay

To determine the effect of extracts on cellular adhesion and morphology after incubation for 48 h, cells were fixed in 4% paraformaldehyde for 15 min, stained with 0.05% crystal violet solution for 30 min and randomly photographed under Nikon TS100 Eclipse microscope (×200 magnification). To quantify the number of attached cells, crystal violet was dissolved with 70% ethanol and absorbance at 570 nm was measured [17].

### 2.10. Membrane Fluidity

The influence of extracts on cell membrane fluidity was investigated with Laurdan labeling. After incubation with extracts, cells were incubated with 2 µM Laurdan for 30 min, and then the fluorescence (440 nm and 490 nm) with the excitation wavelength 370 nm was measured [18,19]. Obtained data were interpreted with generalized polarization (GP) and calculated according to the formula:

(3) $$GP=\frac{I_{440}-I_{490}}{I_{440}+I_{490}},$$

where I_440_ is fluorescence intensity at 440 nm; I_490_ is fluorescence intensity at 490 nm.

### 2.11. Quantitative VEGF Immunoassay

Secreted VEGF protein levels were analyzed by VEGF Human ELISA Kit (Life Technologies). After seeding in culture medium for 24 h, the medium was changed to starving conditions for additional 24 h; then extracts were added for another 48 h. Finally, culture supernatants were collected and VEGF levels were determined according to the manufacturer’s instructions.

### 2.12. Gene Expression Analysis

Total RNA was extracted from cell culture after 48 h incubation with extracts in starving medium using GeneMatrix Universal RNA Purification Kit (Eurex Ltd., Gdansk, Poland) according to the manufacturer’s procedure. RNA samples were purified with Amplification Grade DNase I and reverse transcribed with NG dART RT Kit (Eurex Ltd., Gdansk, Poland). Real time RT-PCR was carried out using SG qPCR Master Mix (Eurex Ltd., Gdansk, Poland) on a BioRad CFX96 qPCR System (Bio-Rad, Hercules, CA, USA). Complementary DNA representing 6 ng of total RNA per sample was subjected of 25 to 40 cycles of PCR amplification. Samples were first incubated at 95 °C for 40 s; then at 55 °C for 30 s; and finally at 72 °C for 30 s. To exclude non-specific products and primer-dimers, after the cycling protocol, a melting curve analysis was performed by maintaining the temperature at 52 °C for 2 s, followed by a gradual temperature increase to 95 °C. The threshold cycle (Ct) values for that gene did not change in independently performed experiments. The level of target gene expression level was calculated as 2^−ΔΔCt^, where ΔΔCt = [Ct(target) − Ct(*β*-actin)]sample − [Ct(target) − Ct(*β*-actin)] calibrator.

Gene expression was normalized using constitutively expressed hypoxanthine phosphoribosyltransferase 1 (*HPRT1*) as a reference gene. The following primer sequences were used to determine the genes’ expression: *MMP2* 5′-CTCATCGCAGATGCCTGGAA-3′ (F) and 5′-TTCAGGTAATAGGCACCCTTGAAGA-3′ (R); *MMP9* 5′-ACGCACGACGTCTTCCAGTA-3′ (F) and 5′-CCACCTGGTTCAACTCACTCC-3′ (R); *ERα* 5′-ATGGAATCTGCCAAGAAGACT-3′ (F) and 5′-GCGCTTGTGTTTCAACATTCT-3′ (R); *ERβ* 5′-CGATGCTTTGGTTTGGGTGAT-3′ (F) and 5′-GCCCTCTTTGCTTTTACTGTC-3′ (R); *VEGF* 5′-CCAGCAGAAAGAGGAAAGAGGTAG-3′ (F) and 5′-CCCCAAAAGCAGGTCACTCAC-3′ (R); *E-cadherin* 5′-CGCATTGCCACATACACTC-3′ (F) and 5′-TTGGCTGAGGATGGTGTAAG-3′ (R); *N-cadherin* 5′-AGTCAACTGCAACCGTGTCT-3′ (F) and 5′-AGCGTTCCTGTTCCACTCCAT-3′ (R); *HPRT1* 5′-TGACCAGTCAACAGGGGACA (F) and 5′-AAGCTTGCGACCTTGACCAT-3′ (R). Data were obtained from at least three independent experiments.

### 2.13. Western Blot Analysis

After the treatment cells were lysed with M-PER Mammalian Protein Extraction Reagent with Halt proteases inhibitor (ThermoFisher Scientific, Waltham, MA, USA). Equal aliquots (20 µg) of the protein samples were separated by 4–12% SDS-PAGE, transferred to nitrocellulose membranes (BioRad, Hercules, CA, USA) and blocked with 5% BSA in TBST buffer. Membranes were incubated with rabbit *β*-actin (Cell Signaling Technology, Danvers, MA, USA) and ERα/ERβ (ThermoFisher Scientific) antibodies at 4 °C overnight, after which they were incubated with horseradish peroxidase-conjugated mouse anti-rabbit secondary antibody (Cell Signaling) for 1 h at room temperature. Western blots were developed using SuperSignal™ West Pico Chemiluminescent Substrate (ThermoFisher Scientific) and quantified by densitometry.

### 2.14. Estrogenic Activity by E-Screen Assay

Estrogenic activity was measured by E-screen assay, where MCF-7 cells were incubated in the presence of 10 nM 17 *β*-estradiol (control cells) or *T. pratense* extracts at 5 µg/mL dosage (<IC_0_) for 48 h. Then cells were fixed in 4% paraformaldehyde for 15 min and stained with a 0.05% crystal violet solution for 30 min. Dye was dissolved with 70% ethanol and absorbance (A) at 570 nm was measured. Relative proliferative estrogenic effect (*RPE*) was calculated according to the formula:

(4) $$RPE\;\left[\%\right]=\;\frac{A\;\mathrm{cells}\;\mathrm{with}\;\mathrm{extracts}}{A\;\mathrm{control}\;\mathrm{cells}}/A\;\mathrm{cells}\;\mathrm{with}\;17\;\beta -\mathrm{estradiol}\;\times \;100.$$

### 2.15. Statistical Analysis

All data are presented as means ± SDs from at least three independent experiments. All obtained results were subjected to statistical analysis. For biological studies, determination of average values and one-way ANOVA analysis followed by the Dunnett’s test were performed using GraphPad Prism 6.0 software (GraphPad Software, Inc., La Jolla, CA, USA) at the significance levels of * *p* ≤ 0.05, ** *p* ≤ 0.01, *** *p* ≤ 0.001.

## 3. Results and Discussion

### 3.1. Isoflavone Compounds Profile and Content

The phenolic composition of red clover (*Trifolium pratense* L.) sprouts was described in detail previously in [10,12,13]. The isoflavone content in CU, CUH, CUF and CUFH extracts was determined by the LC-ESI-MS method and is summarized in Table 1. The total isoflavone content varied from 480.5 to 2751.8 mg/100 g of dry mas of sprouts. Nine phenolic compounds in the CU have been identified: glycosylated forms of isoflavones (daidzin, ononin, genistin and sissotrin) and isoflavones (daidzein, biochanin A, genistein, formononetin and coumestrol). Among samples tested, the CUH extract contained the lowest level of polyphenolic compounds, lower than CU due to glucose cleavage, whereas much higher concentrations of individual isoflavones were determined in sprouts after their lactic fermentation (CUF and CUFH). The hydrolysis process allowed degradation of glycosylated forms of isoflavones leading to the significant elevation of corresponding aglycones, especially before fermentation. The extracts from sprouts treated by lactic fermentation had the highest amounts of formononetin, biochanin A and genistein; thus, they are rich sources of biologically active isoflavones used for production of functional foods and dietary supplements. Due to methoxylation of aglycones biochanin A and formononetin, which are 4′-*O*-methylated plant precursors to genistein and daidzein, they are quickly absorbed into the bloodstream from the gastrointestinal tract and may directly reveal biological activity against cells [10].

### 3.2. Assessment of Trifolium pratense L. Extracts on Cellular Viability

First the effect of extracts’ concentrations from 1 to 15 mg/mL (mg of isoflavones present in dry mass of sprouts/mL) on cellular viability was studied after 48 h incubation. As it is presented in Figure 1, the metabolic activity decreased with increasing extract concentrations starting from 5–7.5 µg/mL depending on the cell line type. The most sensitive were MDA-MB-321 cells, while the most resistant were HUVECs. The highest toxicity against cells revealed itself for CUF and CUFH, where 15 µg/mL dosage decreased MDA-MB-231 cellular activity to 25% compared to the control cells. CU extract had the least influence on metabolic activity: the same dosages decreased MDA-MB-231 and MCF-7 cells’ viability to 50%, whilst the highest concentration tested on HUVEC was able to decrease cells viability to 80%. CUFH contained the highest amount of identified isoflavones aglycones. The highest noncytotoxic concentrations (IC_0_) selected for further studies, and IC_50_ values (the chemical concentration required to reduce the cell activity to 50% of the control), are summarized in Table 2. According to IC_50_ values, the cytotoxicities of *T. pratense* extracts are ranked as follows: CUFH ≥ CUF > CUH > CU. Biologically effective red clover isoflavones’ dosages do not exceed concentrations presented for soy isoflavones (IC_0_ ≈ 12.5–50 µg/mL) [20], which in turn indicates *T. pratense* isoflavones’ biological potential.

The observed *T. pratense* biological activity may be related with the potential of the main identified phenolic constituent: formononetin. There are many studies showing formononetin antiproliferative potential against different cancer cell lines [21], emphasizing its selective activity against ER-positive breast cancer cells, i.e., MCF-7, and the lack of effectiveness in the case of ER-negative MDA-MB-231 cells [22]. In spite of this, Chen et al. demonstrated its proliferation inhibition and apoptosis induction in MCF-7 and MDA-MB-231 cells, with, however, lower efficiency against HUVECs [23]. Authors suggested that observed cellular selectivity was connected with presence of a feedback loop involving miR-375, ras dexamethasone-induced 1 (RASD1) and ERα in HUVECs, but not in MCF-7 cells.

**Figure 2:**
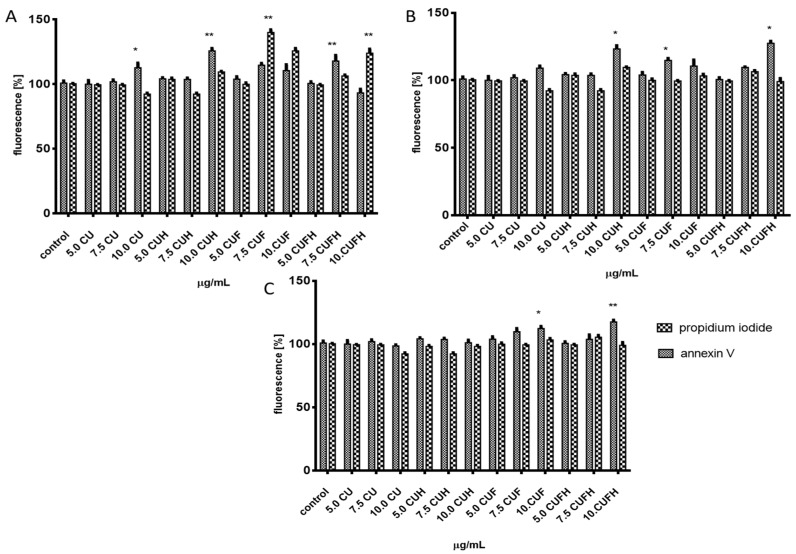
The influence of 48 h of exposure of *T. pratense* extracts on phosphatidylserine externalization on the outer membrane leaflet of apoptotic cells and membrane permeabilization detected with Annexin-V in MDA-MB-231 (**A**), MCF-7 (**B**) and HUVEC (**C**) cells. Values are means ± standard deviations, *n* ≥ 6; statistical significance was calculated versus control cells (untreated); * *p* ≤ 0.05, ** *p* ≤ 0.01.

Due to the cytotoxic activity of elevated concentrations of extracts (Figure 1), we wanted to identify their effects on the type of cellular death induction. Thus, we carried out analysis of putative impact of extracts on apoptosis induction with detection of Annexin V binding to the externalized phosphatidylserine (PS) in the cell membrane (Figure 2A–C). The highest number of apoptotic cells positive for Annexin V staining was observed in MDA-MB-231 line for 7.5 µg/mL dosage of extracts (about 10–20%) (Figure 2A). A prominent level of cells with propidium iodide stained nuclei (about 20–30%), specific for necrosis, was detected for CUF and CUFH at 7.5–10 µg/mL. Propidium iodide is able to enter inside the cells due to the damage of membrane integrity caused by elevated levels of phytocompounds. In MCF-7 cells *T. pratense* at the highest studied concentrations induced apoptotic-type cell death (Figure 2B). Further investigation showed that the most resistant cells were HUVEC (Figure 2C), which is in agreement with the lowest influence of studied preparations on metabolic activity disturbance in that cell line. We want to emphasize that in our studies we had no intention to stimulate cellular death, but we wanted to check the type of cellular death induced under acute influence of extracts. Apoptosis’s induction by red clover extracts would allow the avoidance of damaged cells’ content leakage, thereby being less destructive for neighboring cells [24]. We suppose that observed elevation of propidium iodide fluorescence results from secondary necrosis of apoptotic bodies, which are not engulfed by neighboring cells under in vitro experiments. That experiment allowed us additionally to conclude that *T. pratense* extracts do not disrupt the structure of the cellular membrane, but depending on the number and quantity of isoflavones, they influence the membrane components and its fluidity. There is evidence that *Cicer arietinum* L. extract with isoflavones, mainly biochanin A, formononetin and genistein, induced apoptosis in human breast SKBr3 and MCF-7 cell lines via decreased expression of antiapoptotic Bcl-2 gene and increased expression of apoptosis-promoting gene Bcl-2-associated X protein [25]. The levels of caspase 7, caspase 9, P53 and P21 were elevated. The molecular mechanism of apoptotic induction in MCF-7 cells by genistein is connected with downregulation of ERα and Bcl-2 expression, along with elevation of Bax proapoptotic protein [26].

### 3.3. Trifolium pratense L. as a Chemopreventive Agent

**Figure 3:**
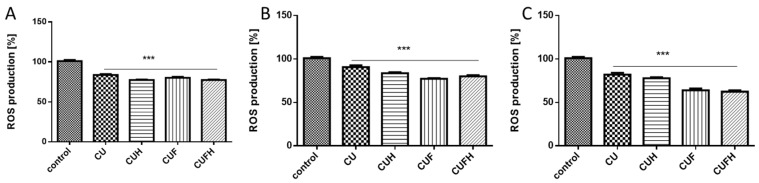
The effect of *T. pratense* extracts on intracellular ROS generation analyzed by DCFH-DA assay after 48 h incubation with MDA-MB-231 (**A**), MCF-7 (**B**) and HUVEC (**C**) cells. Control cells were not exposed to any compound but the vehicle; values are means ± standard deviations from at least three independent experiments; statistical significance was calculated versus control cells (untreated); *** *p* ≤ 0.001.

It is known that polyphenolic compounds possess antioxidant properties allowing the removal and formation of increased reactive oxygen species (ROS). Chronic elevation of ROS induces intracellular oxidative damage leading to metabolic failure and cellular death [27]. Additionally, ROS act within cells to promote migration and influence the behavior of migrating cells through the reorganization of actin cytoskeleton and adhesion [28,29]. Thus, in further steps we checked red clover extracts’ influences at IC_0_ dosages on ROS generation. Cellular antioxidant capacity using a reduced DCF probe takes into account the cellular uptake and metabolism of antioxidants within the cell and reflects the activity of each extract in the biological system. Cells’ pre-incubation with extracts decreased intracellular ROS levels in all cases, with a comparable pattern: the strongest potential revealed CUF/CUFH, and the lowest CU. In human breast cancer cells, the ROS level was lowered by 15–20% compared to the cells treated with the vehicle only (Figure 3A,B). However, the strongest *T. pratense* effect on intracellular oxidative stress reduction was observed in normal HUVEC cells, where extracts obtained from fermented seed sprouts reduced the ROS degree to 70% (Figure 3C).

To evaluate red clover seed sprouts’ chemopreventive properties, we treated cells with *t*-BOOH, which generates oxidative stress under in vitro conditions via production of tertbutoxyl, peroxyl, alkoxyl and methyl radicals catalyzing lipid peroxidation, DNA strand breaks and alteration in intracellular calcium homeostasis [14,16]. As compared to the untreated cells, *t*-BOOH lowered cells metabolic activity by almost 20–30% (Table 3). However, cells’ pre-incubation with the preparations at IC_0_ dosages before *t*-BOOH treatment resulted in a diminishing toxic effect of *t*-BOOH on metabolic activity, by nearly 10–15%.

**Figure 4:**
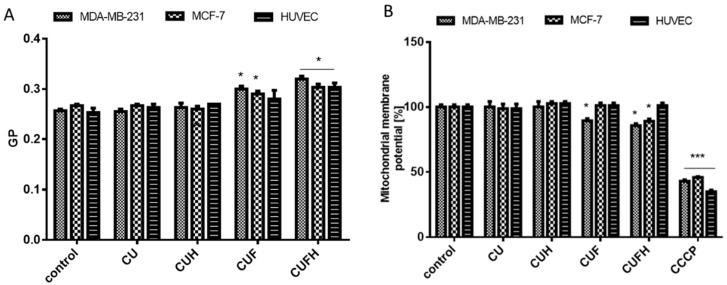
Changes in cell membrane fluidity expressed as values of generalized polarization (GP) for Laurdan probe after incubation of cells with *T. pratense* at IC_0_ (**A**). The effect of *T. pratense* extracts on mitochondrial membrane potential was determined with JC-1 probe. As a positive control for depolarization, carbonyl cyanide m-chlorophenyl hydrazine (CCCP) (50 μM) was used (**B**). control cells were not exposed to any compound but the vehicle; values are means ± standard deviations from at least three independent experiments; statistical significance was calculated versus control cells (untreated); * *p* ≤ 0.05, *** *p* ≤ 0.001.

There are many possible mechanisms of phenolic compounds’ involvement in chemoprevention. One of them is reduction of catalytic activity of enzymes involved in ROS generation; i.e., cyclooxygenase (COX) [30]. Biochanin A is known as strong scavenger of ROS, activator of SOD (superoxide dismutase) enzyme activity and reducer of COX-2 expression [31]. However, there is growing evidence of polyphenols’ reactions with nonpolar compounds present in the hydrophobic inner membrane layer [18,32,33]. Thus, the second way of protection of the cellular membrane against oxidation is membrane stiffening, which resultantly hinders the spreading of newly generated radicals. On the basis of changes in fluorescence intensity of the Laurdan, the membrane generalized polarization (GP) parameters for cells incubated with extracts were determined. Increased values of GP signify increased packing density of the membrane lipid polar heads and decreased membrane fluidity [34]. Current studies clearly indicate that fluidity of cell membranes controls metastatic capacity; thus, decreasing fluidity prevents and inhibits metastasis being a viable therapeutic target [35]. Thus, isoflavones’ interactions with cellular membranes crucially influence cellular anchorage to the ECM or to neighboring cells, but also affect molecular trafficking. Figure 4A shows that extracts enriched with isoflavones (CUF and CUFH) increased the GP value, indicating an increase of membrane stiffening. A comparable mechanism was observed for quercetin, which was able to affect cell membranes’ lipid peroxidation by restoring membrane stiffening [36]. In line with our results are also studies performed by Ajdzanovic et al. demonstrating that soy isoflavones, genistein and daidzein, suppressed metastatic potential of PC cells via decreasing membrane fluidity [19]. The second main *T. pratense* isoflavone, biochanin A, has an impact on membrane stiffening [37].

Because mitochondrial dysfunction can promote progression of a cancer to an apoptosis-resistant/chemo-resistant and/or invasive phenotype, we have studied the effect of extracts on mitochondrial membrane potential (MMP) [16,25]. Though extracts dropped intracellular ROS level, surprisingly, we observed an almost 15% decrease of MMP in MDA-MB-231 cells preincubated with CUF and CUFH at IC_0_ (Figure 4B). Because the reduction of mitochondrial potential and mitochondrial dysfunction are accompanied by damage to the respiratory chain and overproduction of ROS, we suppose that extracts constituents antioxidative properties prevented spreading of radicals. That mode of action was demonstrated for soya beans and flax; see aglycon rich extracts [32]. They were able to decrease mitochondrial membrane potential, and the activities of main radical scavenging enzymes, SOD (superoxide dismutase) and GPx (glutathione peroxidase), leading to the induction of apoptosis in MCF-7 and MDA-MB-231 cells.

### 3.4. The Effect of Trifolium pratense L. on Migration and Attachment of Cells

**Figure 5:**
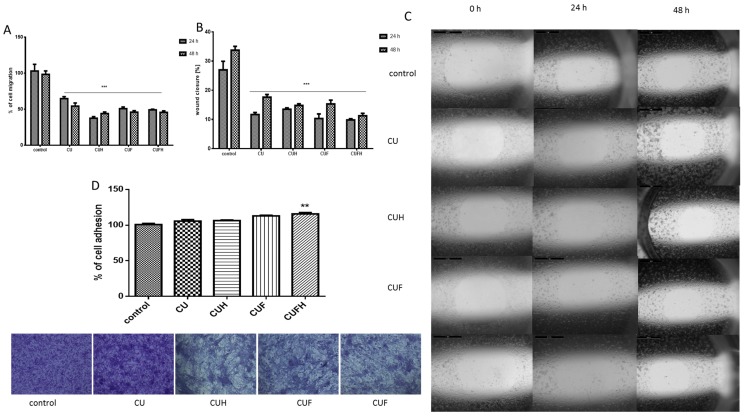
The influence of 48 h of exposure of *T. pratense* extracts on the migration (**A**) and wound healing (**B**) of MDA-MB-231 cells. Migration rates of MDA-MB-231 cells incubated with extracts at IC_0_ dosages into the free detection zones were photographed (×8) (**C**). Cell adhesion to the substrate was measured after staining with crystal violet; randomly chosen fields were photographed at ×200 (**D**). Control cells were not exposed to any compound but the vehicle; values are means ± standard deviations from at least three independent experiments; statistical significance was calculated versus control cells (untreated); ** *p* ≤ 0.01, *** *p* ≤ 0.001.

It is known that in breast cancer metastasis and tumor formation, the migration of cells is implicated [38]. To determine whether red clover preparations are involved in that process, we adopted the scratch wound assay. In case of MDA-MB-321 cells, which are considered a cellular model of breast carcinoma with highly invasive properties [6], all extracts at IC_0_ concentrations not only decreased the migration of cells, but also the rate of wound healing (Figure 5A–C). Both parameters decreased nearly 50% for all extracts. Zhou et al. demonstrated that inhibition of MDA-MB-231 cells’ migration at nontoxic concentrations of formononetin was connected with reduced expression of metalloproteinases MMP-2 and MMP-9, and increased expression of these enzymes inhibitors: TIMP-1 and TIMP-2 [28].

Data perceived with crystal violet staining revealed that the migration inhibition did not result from cellular detachment from the substrate, or the change of cellular shape; instead CUFH even slightly enhanced the number of cells or contacts between cells (Figure 5D). The adhesiveness and attachment of the cells is reciprocally correlated with increased membrane fluidity; thus, agents able to reduce membrane fluidity are potent antimetastatic drugs [31,37,39,40]. Cellular membrane stiffness induced by isoflavones seems to be partially involved in migration and invasiveness limitation. However, it should be taken into consideration that promotion of adhesiveness is desirable at the site of initial dissemination of tumor, because it is unwanted in distant secondary metastasis.

**Figure 6:**
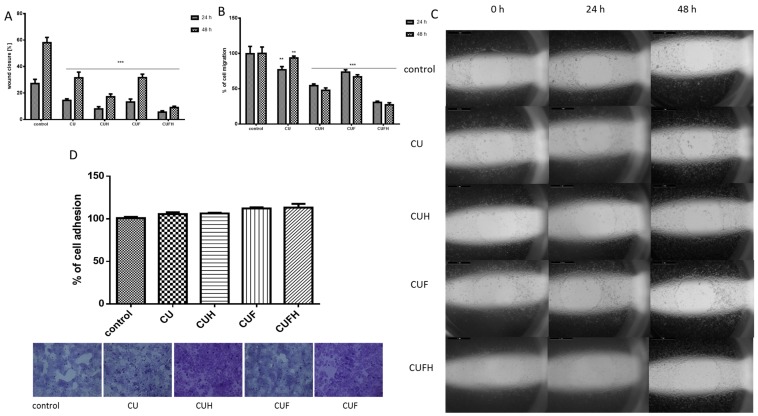
The influence of 48 h of exposure of *T. pratense* extracts on the wound healing (**A**) and migration (**B**) of MCF-7 cells. Migration rates of MCF-7 cells incubated with extracts at IC_0_ dosages into the free detection zones were photographed (×8) (**C**). Cell adhesion to the substrate was measured after staining with crystal violet; randomly chosen fields were photographed at ×200 (**D**). Control cells were not exposed to any compound but the vehicle; values are means ± standard deviations from at least three independent experiments; statistical significance was calculated versus control cells (untreated); ** *p* ≤ 0.01, *** *p* ≤ 0.001.

In the next step, comparable analyses were performed for a known non-invasive breast cancer cellular model—MCF-7 cells (Figure 6). These cells were able to migrate without the addition of any factors inducing invasive characteristics (i.e., 12-*O*-tetradecanoylphorbol-13-acetate (TPA)) [41]. We observed that all extracts inhibited the migration of cells without affecting their detachment; however, extracts’ influences were differentiable: the highest activities were observed for CUH and CUFH preparations, which were able to lower migration of MCF-7 cells by ca. 50% and 65%, respectively.

**Figure 7:**
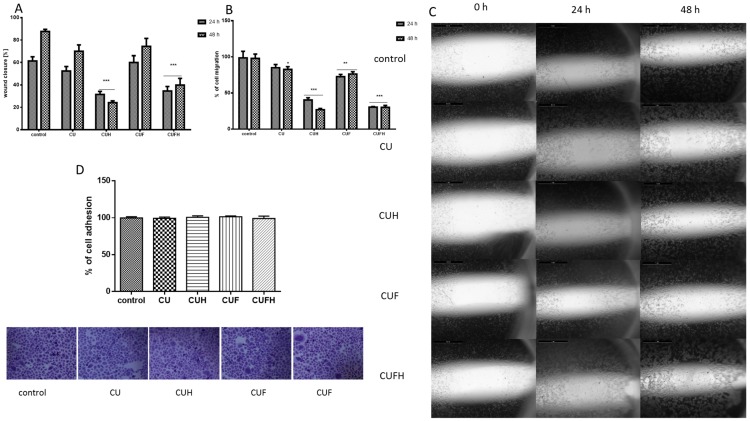
The influence of 48 h of exposure of *T. pratense* extracts on wound healing (**A**) and migration (**B**) of HUVEC cells. Migration rates of HUVEC cells incubated with extracts at IC_0_ dosages into the free detection zones were photographed (×8) (**C**). Cells adhesion to the substrate was measured after staining with crystal violet; randomly chosen fields were photographed at ×200 (**D**). Control cells were not exposed to any compound but the vehicle; values are means ± standard deviations from at least three independent experiments; statistical significance was calculated versus control cells (untreated); * *p* ≤ 0.05, ** *p* ≤ 0.01, *** *p* ≤ 0.001.

The analogous pattern of red clover sprouts’ activity was detected for normal endothelial HUVEC cells. As it is seen in Figure 7, the rates of migration and wound healing were effectively decreased after cells incubation with CUH and CUFH extracts. In these cases, the cellular migration percentage did not exceed 30%; compared to the control, the effect was sustained after 48 h of incubation with the extracts. Data show that the second main *T. pratense* isoflavone, biochanin A (100 µM), was able to inhibit cells’ VEGF-induced migration, but also endothelial nitric oxide synthase (eNOS) participating in the migration and proliferation of endothelial cells during vascular remodeling and angiogenesis [34]. Due to the fact that endothelial cells’ proliferation and migration are essential in the process of angiogenesis, especially for metastasis and sold tumor formation, our results show antimetastatic activity against breast cancer cells and angiogenesis. But it needs to be taken into consideration that detected inhibition of endothelial cells migration may contribute to impairment in the wound healing process, or deepen diabetic vascular complications as a side effect.

On the other hand, we observed that CUF preparation was the weakest inhibitor of endothelial cell migration. Compared to the control cells, CUF decreased migration and wound closure only by 5–15% after 48 h of incubation. That result was unexpected, because CUF and CUFH contained comparable quantities of isoflavones; however, the biological effects were opposite. Among those isoflavones identified in the extracts, formononetin was present in the largest amount; however, its concentration was almost four times higher in extracts obtained after sprouts fermentation—CUF and CUFH. Studies performed by Li et al. identified formononetin as an inducer of endothelial cell migration via recruiting of ERα/ROCK-II (Rho-associated protein kinase) activated complex formation [38]. In signal transduction, ROCK kinase plays a crucial role in cytoskeleton regulation by phosphorylating the myosin-binding subunit of myosin light chain phosphatase, leading to F-actin stress fibers’ formation and focal adhesions, allowing cell migration [38]. Thus, adverse effects observed for extracts suggest the presence of another, not yet identified compound in CUF or CUFH, possessing strong biological activity as an activator or inhibitor; or there is competition between isoflavones with different affinities for binding to ERα and ERβ. Such an effect is the final result obtained after differentiating signaling activation through homo or heterodimerization of receptors [6]. Also, we cannot exclude that the presence of different isoflavones at different concentrations influences the binding affinity to ERs, and thus activates different cellular responses [12].

It was mentioned previously that during cell migration, the actin cytoskeleton undergoes dynamic changes. Among the key promoting intermediators of migration are ROS, especially H_2_O_2_, which can be generated by enzymes known as nicotinamide adenine dinucleotide phosphate (NADPH) oxidase (NO_X_) complexes, and molecules interacting with CAsL (MICAL) proteins [28,29]. ROS have been linked directly to reorganization of filamentous actin structures via oxidation of both cytoskeletal and nuclear actin, or cofilin-1 increasing actin polymerization. Thus, the observed effect of intracellular ROS level lowering by red clover isoflavones can be connected with the inhibition of G-actin or cofilin-1 oxidation, and finally, through blocking F-actin polymerization, decreased actin reorganization of the cytoskeleton and cell movement [13]. Simultaneously, that effect can be deepened by membrane stiffening caused by red clover isoflavones. These conclusions are in agreement with our previous studies, where a docking simulation and isothermal titration calorimetry (ITC) identified interactions of red clover sprouts isoflavones with actin molecules at the ATP binding site reducing its ability to form filaments [13]. Among the isoflavones studied here, the most significant conformational changes of actin were induced by formononetin and biochanin A.

### 3.5. The Influene of Trifolium pratense L. on Gene Expression

**Figure 8:**
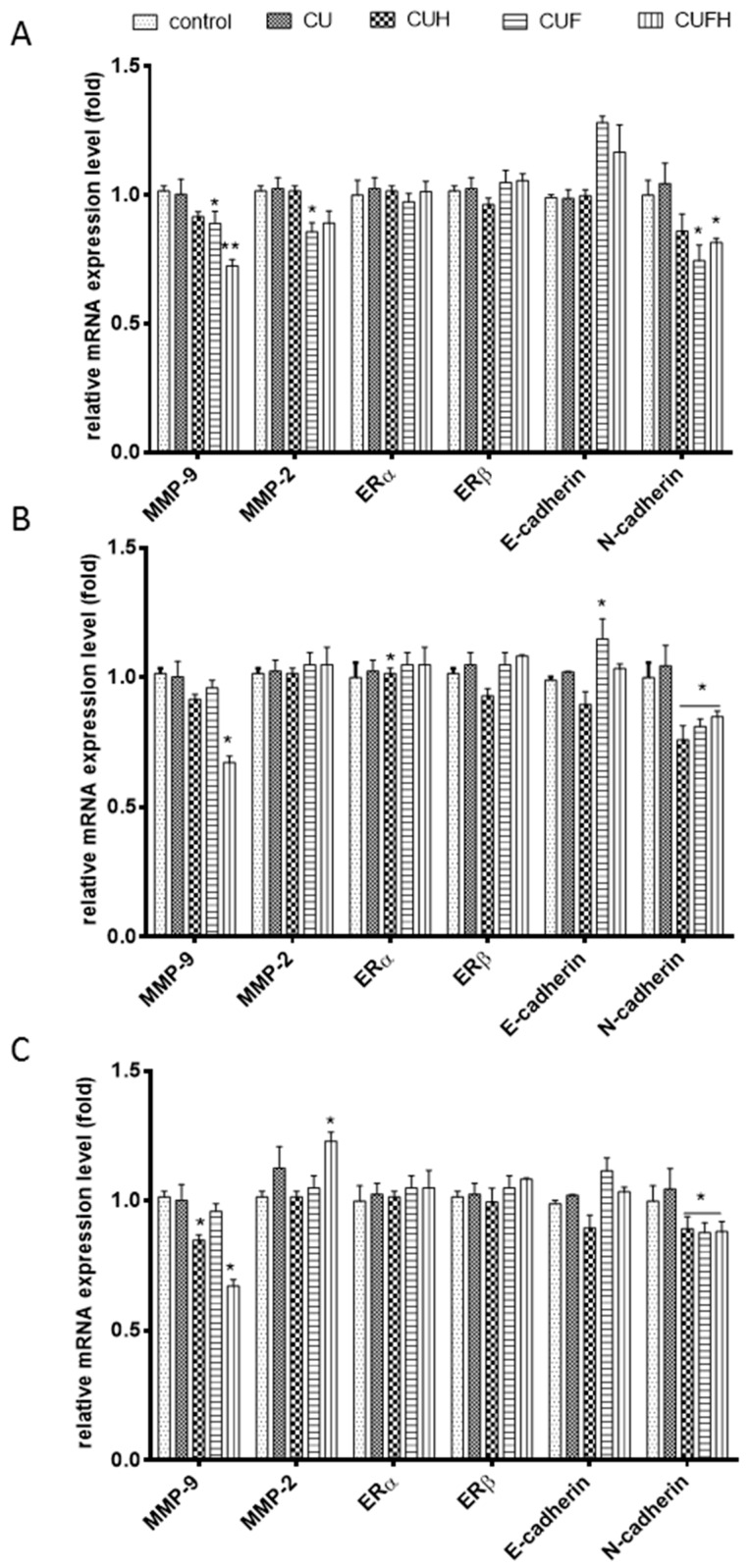
The influence of 48 h of exposure to *T. pratense* extracts on the expression of genes in MDA-MB-231 (**A**), MCF-7 (**B**) and HUVEC (**C**) cells. The expression levels of *MMP-9*, *MMM-2*, *ERα*, *ERβ*, *E-cadherin*, and *N-cadherin* were quantified by real-time PCR and normalized using hypoxanthine phosphoribosyltransferase 1 (*HPRT1*) as a reference gene. Control cells were not exposed to any compound but the vehicle; values are means ± standard deviations, *n* ≥ 3; statistical significance was calculated versus control cells (untreated) * *p* ≤ 0.05, ** *p* ≤ 0.01.

Classical studies have revealed that cell attachment may be correlated with the expression of calcium dependent transmembrane proteins known as cadherins. One of them, E-cadherin, was identified as initiator of contact between cells whose deletion stimulates migration and invasion of breast cancer cells [42]. In this work we found that CUF and CUFH extracts upregulated the mRNA level of E-cadherin in MDA-MB-231 cells, indicating their enhancement of cell-cell type adhesion (Figure 8), whereas in other cell types no effect was observed. Studies performed on human glioma U87MG cells confirmed that formononetin (100 µM) through elevation of E-cadherin protein expression inhibited malignant progression [43]. In contrast to E-cadherin, upregulation of N-cadherin is found in invasive and motile cells [44,45]. Our results showed that in all cell types, the mRNA level of N-cadherin was reduced by CUH, CUF and CUFH, and fermented extracts were the most potent expression regulators.

Observed suppression of cells migration allowed us to suspect that red clover isoflavones restrained invasiveness of breast cancer cells, especially for the MDA-MB-231 cell line. Cancer invasiveness and metastasis are connected with increased expression and activity of enzymes able to degrade and remodel extracellular matrix (ECM) components, such as matrix metalloproteases (MMPs) [22,30]. The key proteolytic enzymes expressed in tumor progression and metastasis are MMP-2 (gelatinase-A) and MMP-9 (gelatinase-B) involved in the degradation of type IV collagen. Thus, next we determined the extracts influence on MMP-2/9, and as it is seen in Figure 8, CUH, CUF and CUFH downregulated *MMP-9* mRNA expression level in all studied cell types. Simultaneously, there was no effect on *MMP-2* mRNA expression. Our observations revealed only the metalloproteinases’ regulation at the transcriptional level and we are aware that extracts’ influences on gelatinolytic enzymes’ activities directly via zymography or level of their secretion are not in this report. What is more, in the regulation of MMP activity are endogenous tissue inhibitors of metalloproteinases (TIMPs); and the quantitative ratio between MMPs and TIMPs expression finally determines the activities of the proteases [22]. TIMP-1 is known to regulate preferentially, MMP-9 activity, whereas TIMP-2 controls MMP-2 activity. Indeed, studies performed with formononetin showed that in downregulation of MMPs’ activities in MDA-MB-321 and 4T1 breast cancer cells are involved elevation of TIMP-1 and TIMP-2 proteins expression, which was followed by suppression of Akt and PI3K kinase phosphorylation. The expression of MMPs can be also regulated at the transcriptional level by different transcriptional factors, such as nuclear factor-kappa B (NF-κB) [46]. Studies with red wine polyphenols demonstrated their ability to reduce in vitro endothelial cell tube formation and migration in wound healing assays, which was followed by decrease of MMP-9 protein release and gelatinolytic activity, ROS level reduction and the activation of the redox-sensitive transcription factor nuclear factor (NF)-κ. There is evidence that formononetin induced HUVEC cells migration via ERα mediated activation of MMP-2/9 proteinases [38]. In our study, we observed a slight elevation of *MMP-2* mRNA level after incubation with CUFH; however, it was followed by decrease of *MMP-9*.

**Figure 9:**
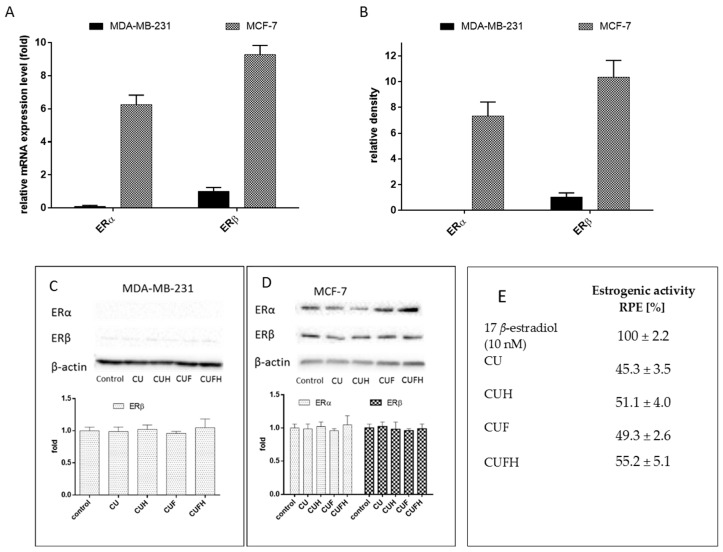
Analysis of relative ERα and ERβ expressions at the mRNA (**A**) and protein levels (**B**) in MDA-MB-231 and MCF-7 cells (samples were calibrated by MDA-MB-231 ERβ). The influence of 48 h of exposure to *T. pratense* extracts on the expression of estrogen receptor proteins in MDA-MB-231 (**C**) and MCF-7 (**D**) cells—bands of western blot representative experiment. The expression levels of ERα and ERβ were quantified by western blot and normalized using β actin as a reference protein. Control cells were not exposed to any compound but the vehicle; values are means ± standard deviations, *n* = 3. (**E**) Relative proliferative effects (RPE) of extracts and 17 *β*-estradiol on proliferative estrogenic activity (RPE) properties according to E-screen assay (*n* ≥ 8).

Next, we assessed the correlation between cellular migration inhibition by *T. pratense* and its influence on cells with expressed differentially estrogen receptor ERα and ERβ isoforms. These proteins belong to nuclear estrogen receptor family of steroid hormone receptors known as regulators of transcription factors are able to modulate target gene transcription by binding as homo and/or heterodimers to estrogen responsive sequences in target gene promoters [1]. ERα and ERβ proteins isoforms, as well as their several alternative splicing variants, share common structural and functional domains and bind estrogens with similar affinity; however, they have different tissue distributions and transcriptional targets. Activation of ERα, especially in the breast, results in induction of cell cycling and stimulation of cell growth, and can result in the initiation and development of cancer. Overexpression of ERβ balances ERα response and was found to inhibit MCF-7 cell proliferation by repressing *c-myc*, *cyclin D1* and *cyclin A* gene transcription, and increasing the expression of p21 and p27 protein, leading to G(2) cell cycle arrest [47]. Estrogens are implicated in bone regeneration and osteoblasts’ antiapoptotic activity stimulation in peri- and postmenopausal periods; thus, the presence of the ERβ and ERα isoforms have functional implications for ligand binding and cellular response. In this regard it is important to identify chemicals able to stimulate bone tissue mineralization without estrogen-induced tumorigenesis or metastasis. There are many studies revealing the isoflavones properties resembling phytoestrogens activities. What is more, some isoflavones may selectively bind to ERβ, indicating a lower affinity for the ERα [12]. Our previous results of ITC modelling and docking simulation with red clover sprouts extracts clearly showed their higher affinity to ERβ than to ERα, indicating their high potential to advantageously activate ERβ without leading to breast cancer cell proliferation. That effect is mainly caused by the presence of formononetin, the predominant isoflavone of the red clover sprouts, which has high affinity for estrogen receptors compared to other red clover aglycones. In our further studies we want to examine *T. pratense* extracts’ influence on ER expression in different cells. The MCF-7 cell line is reported as an ER-positive cellular model, whereas MDA-MB-231 is an ER-negative one (but with positive vimentin expression, which along with N-cadherin, is found in invasive and motile cells) [48]. Human endothelial HUVEC cells are known to express both ER isoforms [48]. Surprisingly, research presented by us revealed that all of the cell lines express *ERα* and *ERβ* mRNA (Figure 8). That observation is in agreement with data obtained in other laboratories, where breast cancer cell lines used in research as being ER-negative expressed not only *ERα* and/or *ERβ* mRNA, but also protein [49]. Hence the presence of the ERβ and ERα isoforms has functional implications for cellular signal transduction after isoflavones binding with ERs. Due to the fact that mRNA expression level in not adequate to provide the pathway of polyphenolic action mechanism, we checked the relative levels of mRNA and protein expression of nuclear estrogen receptors in MDA-MB-231 and MCF-7 cells. As it is seen in the Figure 9A,B, MCF-7 cells expressed more abundantly, both receptor isoforms. What is more, the expression of ERβ receptor exceeded expression of ERα at transcriptional and protein levels. Nevertheless, in case of MDA-MB-231 cells, despite the *ERα* presence on the mRNA level, there was no ERα protein detected (Figure 9A–C), which suggests that in observed cellular signaling ERα is rather not involved, but ERβ as signal transducer may be implicated. Still, comparison of both cell lines relative levels of ERα/β expression shows that nuclear estrogen receptors are rather insignificantly involved in the MDA-MB-231 cellular response to red clover isoflavones, but they may be involved in other non-genomic intracellular action.

These results are consistent with data presented in other studies, where a low-invasive MCF-7 cell line reported as an ER-positive cellular model expressed both types of estrogen receptors, whereas highly-invasive MDA-MB-231, known as ER-negative one, expressed ERβ [50,51]. As it is shown in Figure 8; Figure 9C,D, red clover extracts revealed no significant influence on *ERβ* and *ERα* mRNA expression and protein levels. That allowed us to suppose that observed migration inhibition is not regulated at the transcription level of receptors, but rather by isoflavones binding to the ERβ receptor. Studies performed with PC-3 cells revealed that ERβ-selective agonist DPN decreased the expression of *N-cadherin* [52] which is consistent with what was observed by us—downregulation of *N-cadherin* in the presence of isoflavones. Because ERβ activation was also related with the induction of apoptosis in PC-3M cells, apoptotic cell death may result not only from cytotoxic activity of extract components, but also could be used as functional link for agonistic aglycons binding to ERβ protein [53]. On the other hand, studies performed on the submandibular gland of the rat (an extensive postnatal development model) revealed that by biochanin A was involved in ERβ expression up-regulation [54]. Since estrogenicity is connected with cell proliferation, we evaluated indirectly estrogenic-like effects of *T. pratense* phenolics at dosages lower than IC_0_ through the measurement of MCF-7 cells with crystal violet staining. Figure 9C presents the ratios of the proliferative effects of extracts and 17 *β*-estradiol described by the RPE parameter, where total agonist induces RPE between 80% and 100%, partial agonists induce 25% to 80% and a weak agonist induces between 10% and 25% [55]. Results obtained for preparations are comparable to each other and lower than 80%, which suggests that red clover extract components may act as partial agonists to ERα, and are therefore able to antagonize the effects of estrogens in ERα-positive breast cancer cells. *T. pratense* preparations contain large quantities of formononetin, and that compound was shown to reveal dosage-dependent estrogenic activity in MCF-7 cells [55]. Still, it should be taken into account that, as observed in the estrogenic activity assay, increased MCF-7 cell proliferation may result not only from the activation of estrogen receptors, but mediated signaling pathways as well [3,4].

Recently, apart from classical estrogen receptors ERα and ERβ, the orphan receptor GPR30 (GPER-1)—also able to bind estrogen, has been identified [40]. That protein is a membrane G-protein coupled receptor involved in mediation of non-genomic estrogenic and second messenger response [56]. There is evidence that phytoestrogens are able to activate GPR30 leading to several estrogen-dependent disorders, especially breast cancer [57]. GPR30 activation triggers a complex signaling pathway, including epidermal growth factor receptor (EGFR) transactivation, whose overexpression increases proliferation of tumor cells. Literature analysis shows that GPR30 receptors are expressed by all cellular models used in this study: MDA-MB-231, MCF-7 and HUVEC cells [40,57,58]. More detailed studies revealed the antagonistic effect between GPR30 and ERβ signaling in MDA-MB-231 cells, where GPR30 activation increases motility and invasiveness in ER-negative breast cancer cells [59]. These findings highlight the important interplay between different estrogen receptors in estrogen induced cell motility and invasiveness in ER-negative breast cancer cells. In that molecular signaling pattern, genistein is a strong inhibitor of tyrosine kinases and EGFR advancing antimetastatic properties [60]. Thus, genistein behaves as a ligand of ERα, ERβ and GPR30 receptors with binding preference to a single receptor type depending on the compound concentration, clearly exposing interplay and crosstalk between these signaling molecules, as well as genistein’s pleiotropic action. Among other isoflavones with GPR30 agonist activity, coumestrol was also identified, which on the other hand is known as an antagonist of ERα and ERβ receptors. Thus, after its binding to GPR30 and the activation of signaling pathways in breast cancer cells, stimulation of proliferation could be observed [3].

### 3.6. The Effect of Trifolium pratense L. on VEGF

**Figure 10:**
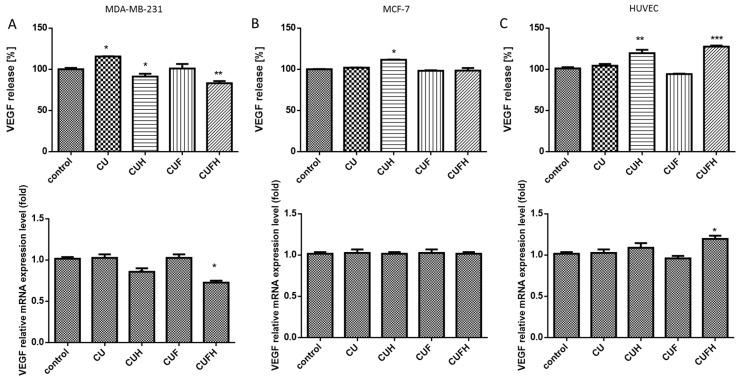
The influence of 48 h of exposure of *T. pratense* extracts at IC_0_ dosages on VEGF secretion and *VEGF* mRNA level in MDA-MB-231 (**A**), MCF-7 (**B**) and HUVEC cells (**C**). Control cells were not exposed to any compound but the vehicle; values are means ± standard deviations, *n* = 3; statistical significance was calculated versus control cells (untreated) * *p* ≤ 0.05, ** *p* ≤ 0.01, *** *p* ≤ 0.001.

Angiogenesis and new blood vessel formation from the preexisting ones is essential for tumor growth and metastasis. The most prominent angiogenic agent markedly induced in breast cancer is VEGF (vascular endothelial growth factor) [61]. In this manner we wanted to check the influence of red clover extracts on VEGF secretion to the culture medium by different types of cells. As it is shown in the Figure 10, significant decreases of VEGF protein and mRNA levels (10–20%) were observed only after MDA-MB-231 cells incubation with CUH and CUFH. Because VEGF stimulates membrane kinase receptor VEGFR2 following endothelial cells activation, diminished VEGF levels in the cellular environment and disruption of VEGF-related cell signaling may result in decreased proliferation, migration and tube formation [62]. In the case of MCF-7 cells, *T. pratense* phytocompounds showed almost a lack of influence in this field, yet still revealed chemoprevention of breast cancer cells. Contrary to results of the same extracts, CUH and CUFH positively affected VEGF protein release (by nearly 20% and 25%, respectively) and the level of mRNA expression (respectively, by 5% and 15%) in endothelial cells. These ambiguous outcomes may suggest the cellular specific response: isoflavones were able to inhibit VEGF release from highly invasive cells, and at the same time stimulate secretion by normal cells. That indirectly indicates that red clover polyphenols would not disturb wound healing process, but nevertheless may promote blood vessel formation tumor growth. Despite these observations, other studies revealed pleiotropic effect of formononetin, which was able to inhibit HUVEC cells angiogenesis by blocking the FGFR2-mediated PI3K-Akt signaling pathways [63]. In breast cancer cells, VEGF is a target gene for ERα or ERβ. It was observed that HUVEC incubation with GPR30 agonist increased proangiogenic VEGF and bFGF contents in supernatant. Thus, while the elevation of VEGF release by HUVECs seems to be transcriptionally regulated by the CUFH component, a contradictory breast cell response leading to a VEGF decrease suggests CUFH to be a GPR30 inhibitor. Studies performed with synthetic GPR30 antagonist showed that after its binding, apoptosis was triggered with Bax, Caspase-3 and Caspase-9 elevation, and that is coherent with MDA-MB-231’s apoptosis induction by CUFH.

## 4. Conclusions

Red clover sprouts are rich source of isoflavones. Extracts obtained from fermented sprouts (CUF and CUFH) contained high amount of isoflavone aglycons in which formononetin, biochanin A and genistein dominated. That chemical composition is very relevant in case of *T. pratense*’s biological influence on human body, because isoflavones present in diet could be transported through the intestinal cell barrier in intact aglycone form [64] and are absorbed faster and in greater amounts than corresponding glucosides [65]. Earlier clinical studies have shown that the maximum recommended level for safe isoflavone intake is 75 mg/day, and a plasma concentration should not exceed 10 µM [66,67]. The concentration of clover extracts used IC_50_ was 5 μg/mL, which corresponds to about 3 μM (calculated with biochanin A), assuming that extracts contained 100% isoflavones. Fermented sprouts’ extracts contained aglycones, in contrast to extracts of sprouts prior to fermentation, in which there were also glycosides poorly absorbed in the body. Methoxy derivatives, such as biochanin A, even as aglycones, may be poorly absorbed; however, the administration of isoflavones in the mixture contributes to an increase in bioavailability, potentially through increased enterohepatic circulation [31]. The extracts influenced biological activities of human breast cancer cell lines MDA-MB-231 and MCF-7 and exerted cytotoxicity against both cells types, whereas in case of ERα-positive MCF-7 cells, dosages below IC_0_ revealed little estrogenic activity of stimulating cell growth. Lack of ERα protein expression in MDA-MB-231 cells may influence their lower resistance to the cytotoxic activities of the isoflavones herein in comparison to ERα-positive cells. Despite these differences, extracts at IC_0_ dosages influenced cellular migration, intracellular ROS generation, membrane stiffening, adhesion and VEGF secretion, and regulated MMP-9, N-cadherin and E-cadherin at the transcriptional level. Simultaneously, isolated phenolics at comparable dosages revealed no toxicity against normal HUVEC cells. That can be connected not only with different cellular type, but also with the potential expression of both ER-receptor isoforms [48].

**Figure 11:**
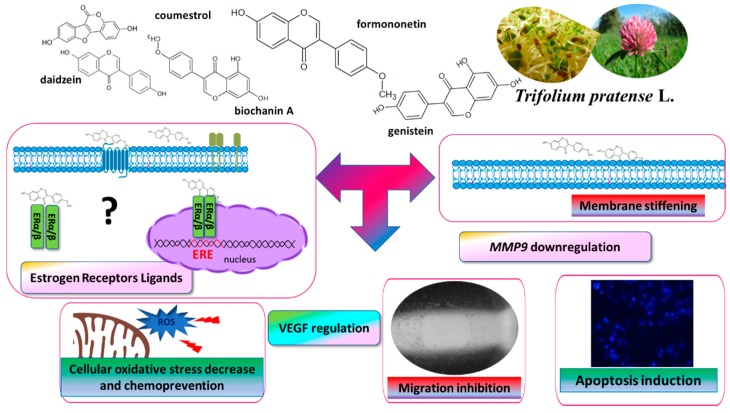
*Trifolium pratense* isoflavones as modulators of cell migration—the proposed mechanism of action. They possess cytoprotective activity against ROS generation, stiffen cellular membrane, induce apoptotic type cell death and influence expression of *MMP9* and *VEGF*. ERα/β—estrogen receptor α/β; ERE—estrogen responsive element; MMP9—matrix metalloproteinase 9; VEGF—vascular endothelial growth factor.

Preliminary results for the cellular models suggest that CUFH isoflavones exhibit anti-metastatic effects in ERα-negative and ERα-positive cells, so we are proposing that these effects were exerted without ERα nuclear estrogen receptor involvement, and *T. pratense* isoflavones intake could be an effective dietary supplement for breast cancer patients, reducing cell migration and invasiveness. However, it should be taken into account that all studied cells revealed the expression of ERβ receptors. What is more, the observed effects may in part result from isoflavones binding with recently identified membrane estrogen receptor GPR30. To investigate possible modulation of ERα, ERβ or GPR30 receptors’ cross talk and transactivation by extract components, more detailed studies should be performed, especially in the presence of selective receptors inhibitors or with transfected cells expressing selectively ERα/ERβ or GPR30 receptors and estrogen-responsive reporter gene constructs to identify red clover isoflavones as agonists or antagonists. Besides, in order to dissect the specific contribution of an extract’s constituents, the effects of isoflavones being separated or in mixtures, in a concentration dependent matter, on cellular proliferation and migration, should be investigated. In summary, we propose a preliminary mechanism accounting for activity of *Trifolium pratense* L. isoflavones (Figure 11); however, these properties need further molecular evaluation: the beneficial effects of extracts after in vitro digestion or obtained after their incubation with gut microflora.

## Figures and Tables

**Table 1:** Isoflavones composition of *Trifolium pratense* L. sprouts (the results are expressed as mg of phenolic compounds per 100 g of dry mass). Phenolic compounds (mg/100 g of dry mass). n.d.—not detected.

Type of Preparation (Name of Extract Studied)	Daidzin	Ononin	Genistin	Sissotrin	Daidzein	Biochanin A	Genistein	Formononetin	Coumestrol	Phenolics Total
Clover/UVB (CU)	2.42	216.38	17.67	2.91	1.22	75.31	3.75	250.39	0.67	570.66
Clover/UVB/H (CUH)	n.d.	n.d.	n.d.	n.d.	2.70	77.16	14.75	385.25	0.67	480.52
Clover/UVB/F (CUF)	n.d.	21.08	0.20	0.45	3.06	265.01	83.77	2377.69	0.59	2751.84
Clover/UVB/F/H (CUFH)	n.d.	n.d.	n.d.	n.d.	3.06	265.29	83.89	2390.83	0.59	2743.67

**Table 2:** The influence of *T. pratense* extracts on cells viability presented as IC_0_ and IC_50_ values. The influence of each compound was measured with PrestoBlue assays; values are means from at least three independent experiments (*n* ≥ 9).

*T. pratense* Extracts	IC_0_ [µg/mL]			IC_50_ [µg/mL]		
	MDA-MB-231	MCF-7	HUVEC	MDA-MB-231	MCF-7	HUVEC
CU	6.2	7.3	10.0	≥15.0	>15.0	>15.0
CUH	6.0	7.0	9.8	14.8	>15.0	>15.0
CUF	5.4	6.0	7.0	9.8	15.0	15.0
CUFH	5.0	5.9	7.1	10.0	15.0	14.8

**Table 3:** The influence of cells’ preincubation with maximal nontoxic extracts concentrations (IC_0_) of *T. pratense* extracts for 48 h on metabolic activity of cells. Oxidative stress was induced by 500 μM *t*-BOOH (2 h), then PrestoBlue assay was performed.

*T. pratense* Extracts	Cells Viability [%]		
	MDA-MB-231	MCF-7	HUVEC
	79.50 ± 3.85	75.25 ± 3.02	71.49 ± 1.08
CU	86.64 ± 2.21	82.48 ± 2.20	80.55 ± 4.32
CUH	88.45 ± 6.14	90.10 ± 3.95	82.14 ± 6.45
CUF	90.21 ± 6.12	89.14 ± 6.45	76.25 ± 6.45
CUFH	90.11 ± 9.42	91.70 ± 4.31	77.36 ± 3.14
